# A Review on Recent Advances in Signal Processing in Interferometry

**DOI:** 10.3390/s25165013

**Published:** 2025-08-13

**Authors:** Yifeng Wang, Fangyuan Zhao, Linbin Luo, Xinghui Li

**Affiliations:** Tsinghua Shenzhen International Graduate School, Tsinghua University, Shenzhen 518055, China; wangyifeng25@163.com (Y.W.); fy-zhao24@mails.tsinghua.edu.cn (F.Z.); luolb24@mails.tsinghua.edu.cn (L.L.)

**Keywords:** optical interferometry, homodyne, heterodyne, hardware platforms

## Abstract

Optical interferometry provides high-precision displacement and angle measurement solutions for a wide range of cutting-edge industrial applications. One of the key factors to achieve such precision lies in highly accurate optical encoder signal processing, as well as the calibration and compensation techniques customized for specific measurement principles. Optical interferometric techniques, including laser interferometry and grating interferometry, are usually classified into homodyne and heterodyne systems according to their working principles. In homodyne interferometry, the displacement is determined by analyzing the phase variation of amplitude-modulated signals, and common demodulation methods include error calibration methods and ellipse parameter estimation methods. Heterodyne interferometry obtains displacement information through the phase variation of beat-frequency signals generated by the interference of two light beams with shifted frequencies, and its demodulation techniques include pulse-counting methods, quadrature phase-locked methods, and Kalman filtering. This paper comprehensively reviews the widely used signal processing techniques in optical interferometric measurements over the past two decades and conducts a comparative analysis based on the characteristics of different methods to highlight their respective advantages and limitations. Finally, the hardware platforms commonly used for optical interference signal processing are introduced.

## 1. Introduction

As a core technology in modern industrial production and scientific research, displacement measurement has consistently played a vital role [1,2,3,4]. From rudimentary mechanical tools to today’s high-precision measurement systems that integrate optics, electronics, and other interdisciplinary innovations, demands on measurement accuracy have evolved from the micrometer scale to the nanometer, sub-nanometer, and even picometer levels [5,6,7]. Driven by advances in sensor technologies and digital manufacturing [8], displacement measurement methods continue to undergo innovation and breakthroughs, offering critical support to a broad range of sectors, including optics [9,10,11,12,13,14,15], biomedical engineering [16,17,18], electronics [19], and manufacturing industry [20,21].

In mechanical manufacturing, precise displacement measurement underpins the machining and assembly of high-precision components, directly influencing product quality and performance [22,23]. For instance, in computer numerical control machining, the characterization of surface parameters, the accurate positioning of cutting tools, and defect detection all depend on continuous improvements in measurement precision [24,25,26]. In the electronics industry, particularly in semiconductor fabrication, displacement measurement in lithography processes must reach nanometer-level accuracy, which is essential to ensuring high integration density and performance of microchips. Specifically, the wafer stage in lithography systems, which is responsible for ultra-precise and high-speed motion, must achieve extremely accurate positioning and motion control to meet overlay and throughput requirements [27,28]. For example, ASML’s TWINSCAN NXE: 3600D EUV lithography system achieves a resolution of 13 nm and an overlay accuracy of 1.1 nm, enabling volume production at 5 nm and 3 nm technology nodes, with sub-nanometer positioning precision for both the wafer and mask stages [29].

With ongoing technological advancements, the requirements for measurement precision, speed, and reliability are becoming increasingly stringent. A variety of displacement measurement methods have emerged and continue to evolve. While traditional contact-based mechanical approaches remain in use [30], non-contact measurement techniques are gaining traction in high-precision manufacturing. These include capacitive sensing [31,32,33], eddy current sensing [34,35,36], ultrasonic methods [37,38], optical triangulation [39,40,41], confocal measurement [42,43,44,45,46,47,48], laser interferometry [49,50,51,52,53], and grating interferometry [54,55,56,57,58,59]. Among them, laser interferometry and grating interferometry, both based on optical interference principles, stand out due to their high precision, resolution, non-contact nature, and strong resistance to interference. As a result, they have become essential tools in ultra-precision machining, metrology, microelectronics manufacturing, and scientific research, where extreme measurement accuracy is required [60,61]. In contrast, laser triangulation, while offering non-contact measurement and rapid response, suffers from resolution degradation over extended ranges due to optical divergence. Capacitive and inductive sensors, relying on field variation, provide stable performance in harsh environments but are limited by short measurement ranges within 10 mm and susceptibility to electromagnetic interference.

Laser interferometry and grating interferometry both utilize optical coherence to extract displacement information [49,62]. They can achieve multi-degree-of-freedom measurement with sub-nanometer or even picometer-level precision [63,64,65,66,67,68], as well as absolute positioning [69,70,71,72,73,74]. A laser interferometer detects target displacement by analyzing phase shifts in the reflected light from a measurement surface relative to a reference beam, enabling a wide dynamic range from sub-nanometer to several kilometers [75]. However, because its measurement reference is the laser wavelength, it is susceptible to fluctuations in the air’s refractive index under non-vacuum conditions and is thus sensitive to environmental factors such as temperature and humidity, requiring compensation and calibration [76,77,78].

In contrast, grating interferometers measure target displacement by detecting the phase shift between diffracted beams from a measurement grating and a reference grating [79,80,81,82,83,84]. Unlike laser interferometers that rely on the wavelength of light as the measurement standard, grating interferometers use the grating pitch as a reference, which renders them inherently less sensitive to environmental variations such as temperature and pressure fluctuations. This characteristic makes them particularly well-suited for high-precision measurements in industrial environments where robustness and stability are critical [85,86,87,88]. The measurement accuracy of grating interferometers, however, is intrinsically dependent on the precision and uniformity of the grating itself. Laser lithography has become the key fabrication method for such gratings [89,90], offering high resolution and patterning accuracy over large areas [91,92,93]. The advancement of high-precision fabrication techniques for two-dimensional gratings [94,95,96,97,98] provides the foundational metrology infrastructure for integrated in-plane and out-of-plane multi-axis measurement systems, enabling sub-nanometer positioning accuracy essential to motion control applications such as wafer stage positioning in semiconductor lithography equipment [99,100]. By optimizing exposure parameters, mask design, and etching processes, it is possible to produce gratings with exceptional pitch accuracy, minimal defects, and high cost-efficiency, thus providing a robust metrological foundation for achieving sub-nanometer measurement precision [101,102,103].

Further improvements in measurement precision increasingly rely on the signal subdivision and processing capabilities of demodulation algorithms [104,105,106]. Additionally, in multi-degree-of-freedom measurements, laser interferometers typically require multiple subsystems arranged around the target, whereas grating interferometers can achieve up to six degrees of freedom within a compact space [107,108,109,110].

Based on their operating principles, optical interferometers are generally categorized into homodyne and heterodyne systems [53,111,112,113]. Homodyne interferometers use a single-frequency laser to generate direct-current (DC) amplitude-modulated signals, from which displacement information is extracted through phase demodulation [107,114,115,116]. These systems are structurally simple and often employed at low speed and high resolution. However, they are highly sensitive to light source stability and environmental noise, which makes them vulnerable to intensity fluctuations and DC drift [62,117,118,119]. In contrast, heterodyne interferometers employ two laser beams with a small frequency offset to produce a beat-frequency signal [56,120,121,122]. The low-frequency envelope enables the extraction of high-frequency phase information that would otherwise be difficult to measure directly. Heterodyne systems are more resistant to interference, offer higher sensitivity, and are better suited for multi-axis synchronous measurements that involve high resolution and speed, which places them among the dominant architectures in contemporary interferometry [123,124,125,126]. Currently, both measurement methods can achieve displacement measurement resolutions at the sub-nanometer or even picometer level [62,68,127,128].

Displacement demodulation algorithms are critical to achieving high-precision measurements in interferometers and directly influence system accuracy and performance. In practical applications, interference signals often suffer from periodic nonlinearity due to mixed-frequency sources and polarization crosstalk, requiring algorithmic compensation and correction during phase extraction [129,130,131,132].

For homodyne interferometers, commonly used demodulation algorithms include error calibration methods [133,134,135,136] and ellipse parameter estimation methods [51,137,138,139,140,141,142,143,144]. Error calibration methods sequentially correct various nonlinear errors to obtain standard orthogonal sine and cosine signals, but they involve relatively high computational complexity. In contrast, ellipse parameter estimation methods fit and estimate the parameters of the Lissajous figure formed by the two orthogonal signals to extract displacement information.

Heterodyne systems adopt more diverse and complex phase demodulation algorithms, including pulse counting [145], quadrature phase-locked techniques [146,147,148,149,150], and Kalman filtering [151]. Pulse-counting methods determine the phase by quantifying the time difference between two signals. They are simple and easy to implement but highly dependent on signal quality and frequency stability and perform poorly under low signal-to-noise conditions. Quadrature phase-locked methods employ digital phase-locked loop techniques to extract amplitude and phase information through mixing and filtering, overcoming the cycle-counting limitations of pulse methods and offering stronger noise resistance [146]. However, their dynamic range and noise performance are influenced by the cutoff frequency of the low-pass filter. Kalman filtering directly estimates the phase parameters from the interference signal, providing high real-time performance and exceptional measurement accuracy, with broad application potential [151].

Alongside algorithmic design, the choice of hardware platform plays an essential role in the overall implementation of optical systems. Common computing platforms include personal computers (PCs) [152,153,154,155,156,157], microcontroller units/digital signal processors (MCUs/DSPs) [158,159,160,161,162,163], field-programmable gate arrays (FPGAs) [133,135,136,151,164,165,166,167,168,169], and application-specific integrated circuits (ASICs). PCs offer powerful computational capabilities and rich software resources, facilitating algorithm development and debugging, but they tend to be bulky and lack real-time responsiveness. MCUs and DSPs have excellent real-time performance, compact size, and low power consumption, which makes them ideal for embedded applications, though their computational power may limit the implementation of complex algorithms. FPGAs provide high parallelism and flexibility for real-time signal processing and can be reconfigured for specific tasks. ASICs, with their high integration, low power consumption, and superior performance, are well-suited for high-end applications requiring compact systems, low latency, and fast measurement speeds.

**Figure 1:**
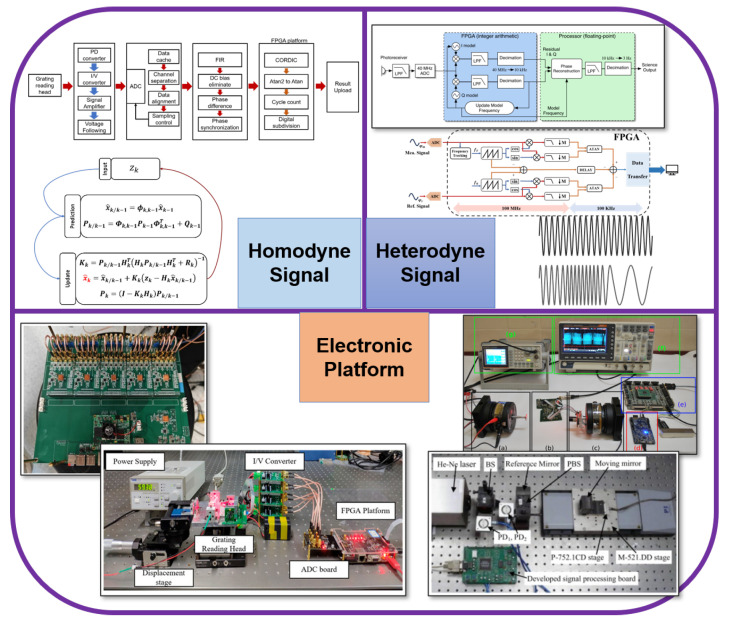
Overall writing framework of this manuscript [133,145,146,147,148,149,150,157,162,170,171].

This review aims to provide a systematic and comprehensive overview of signal processing algorithms for both homodyne and heterodyne optical interferometers, along with their associated computational hardware platforms, as the overall writing framework shows in Figure 1. Section 2 introduces the operating principles and signal characteristics of optical interferometers, laying the foundation for the following algorithmic discussion. Section 3 focuses on demodulation methods for homodyne and heterodyne systems, including their principles, advantages, limitations, and real-world applications. Section 4 surveys commonly used computational hardware platforms, including PCs, MCUs/DSPs, FPGAs, and ASICs, highlighting their architectural features, performance advantages, and practical roles in displacement demodulation and offering comparative insights to guide system selection and design. Through this review, we aim to provide readers with a comprehensive understanding of signal conditioning techniques for optical interferometers and offer valuable guidance for both academic research and engineering practice in related fields.

## 2. Patterns of Optical Interferometric Signals

In the field of optical interferometry, homodyne and heterodyne signals, as the primary signal patterns in coherent detection, play a crucial role in precision measurement systems such as grating interferometers and laser interferometers. A homodyne signal is generated by the interference between signal light carrying information and a local oscillator (LO) light that is strictly matched in frequency and phase [172,173,174,175,176,177,178,179,180]. The resulting signal directly reflects the amplitude and phase of the original optical field, offering ultra-high sensitivity and phase resolution [134,136,167,181]. These characteristics make homodyne detection well-suited for weak optical signal sensing and nanometer-scale displacement measurements. However, this technique imposes stringent requirements on light source stability and optical path consistency, as slight frequency drifts may lead to signal distortion [106,182].

By contrast, a heterodyne signal [183,184,185,186,187,188] is produced by interfering the signal light with an LO light of a different frequency, resulting in an electrical signal at a fixed intermediate frequency (IF), which facilitates subsequent filtering, amplification, and demodulation. It exhibits strong anti-interference capability, and the IF processing can effectively suppress the influence of environmental noise and optical path fluctuations, making it applicable to dynamic measurements in complex environments [148,151,189,190]. Although the detection sensitivity of heterodyne detection is slightly lower than that of homodyne detection, it has lower requirements for frequency synchronization, providing better system robustness and engineering feasibility [191,192,193,194]. Nevertheless, the generation of heterodyne interference signals requires a dual-frequency laser source, which significantly increases the cost.

### 2.1. Homodyne Signal

**Figure 2:**
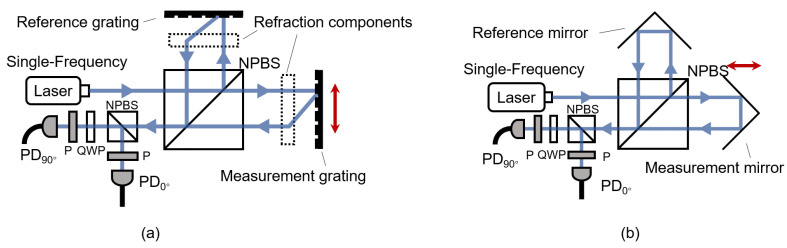
Homodyne grating interferometer and laser interferometer in a Michelson-type structure. (**a**) Homodyne grating interferometer. (**b**) Homodyne laser interferometer.

Homodyne grating interferometry employs a single-frequency laser source. A Michelson-type homodyne grating interferometer is illustrated in Figure 2a. A laser beam of frequency *f* first passes through a non-polarizing beam splitter (NPBS), where it is split into two beams directed orthogonally, which are incident on the measurement and reference gratings, respectively. After being diffracted by the gratings, the beams are collimated and then recombined at the NPBS to produce interference. To determine the direction of motion, the interference beam is further split into two beams by an additional NPBS. One beam passes through a polarizer and is detected by a photodiode (PD), while the other beam passes through a quarter-wave plate (QWP), followed by a polarizer, and is then detected by a second PD. The two detected signals exhibit a 90° phase difference, enabling quadrature signal analysis.

The single-frequency laser beam emitted by the laser source can be expressed as

(1) $$E_{0}=A_{0}cos\left(2\pi ft+\phi_{0}\right)$$

where *f* denotes the laser frequency, $A_{0}$ represents the amplitude, and $\phi_{0}$ is its initial phase. After diffraction by the reference grating, the beam can be written as

(2) $$E_{r}=A_{r}cos\left(2\pi ft+\phi_{r}\right)$$

where $A_{r}$ is the amplitude and $\phi_{r}$ is the phase. The frequency of the diffracted light remains unchanged at *f*. Both the amplitude and phase undergo alterations due to change in the optical path and attenuation by optical components.

When the measurement grating is in motion, the diffracted light from the measurement grating experiences a frequency shift because of the optical Doppler effect. This phenomenon can be mathematically described as

(3) $$\begin{array}{cc} E_{m} & =A_{m}cos\left[2\pi \left(f+\Delta f_{k}\right)t+\phi_{m}\right] \end{array}$$

(4) $$\begin{array}{cc} \; & =A_{m}cos\left[2\pi ft+\Delta \phi_{m}+\phi_{m}\right] \end{array}$$

Here, $A_{m}$ and $\phi_{m}$ denote the amplitude and phase of the measurement beam, respectively. $\Delta f_{k}$ represents the frequency variation caused by the displacement in the measurement grating. This frequency change can be equivalently characterized as a phase change $\Delta \phi_{m}$, which inherently encodes the displacement signal of the measurement grating and exhibits a linear relationship with the displacement. The displacement is calculated using the formula

(5) $$L=k\Delta \phi_{m}$$

where *L* is the displacement in the measurement grating and *k* is a proportionality coefficient determined by the grating parameters and the optical encoder structure.

When the measurement beam and reference beam interfere, the wave equation of the resulting interference light is given by $E_{m}+E_{r}$. Owing to bandwidth limitations, a PD can only respond to low-frequency signals. The signals detected by PD_0°_ and PD_90°_ can be expressed as follows:

(6) $$I_{cos}\propto A_{r}^{2}+A_{m}^{2}+2A_{r}A_{m}cos\left(\Delta \phi_{m}\right)$$

(7) $$I_{sin}\propto A_{r}^{2}+A_{m}^{2}+2A_{r}A_{m}sin\left(\Delta \phi_{m}\right)$$

By deriving the phase difference $\Delta \phi_{m}$ from $I_{cos}$ and $I_{sin}$, the displacement in the measurement grating can be calculated using Formula (5). Therefore, the precise calculation of the phase difference using quadrature signals is critical to homodyne signal processing.

The homodyne laser interferometer also generates homodyne signals, as shown in Figure 2b. Compared with the grating interferometer, the laser interferometer uses a measurement mirror instead of a measurement grating. When the measurement mirror moves, its reflected beam undergoes a frequency shift due to the Doppler effect. The homodyne signal pattern generated by the homodyne laser interferometer is essentially consistent with that of the grating interferometer, as expressed by Formulas (6) and (7). However, the measurement reference in laser interferometry is the wavelength of the light, which makes it more susceptible to environmental influences. In comparison, grating interferometers use the grating pitch as the measurement reference, making them less sensitive to environmental variations.

### 2.2. Heterodyne Signal

**Figure 3:**
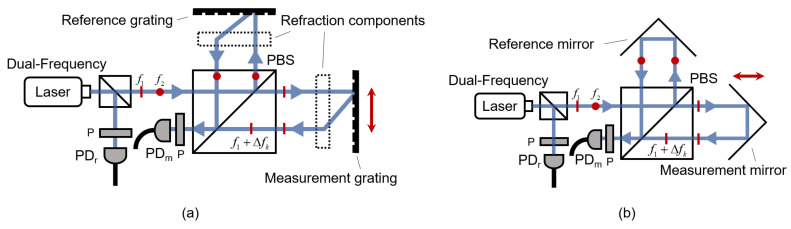
Heterodyne grating interferometer and laser interferometer in a Michelson-type structure. (**a**) Heterodyne grating interferometer. (**b**) Heterodyne laser interferometer.

Heterodyne grating interferometry employs a dual-frequency laser source. Figure 3a illustrates a typical heterodyne grating interferometer. The dual-frequency laser emits two orthogonally linearly polarized beams with frequencies $f_{1}$ and $f_{2}$, which are separated into two beams by an NPBS. The reflected beam passes through a polarizer, interferes, and is detected by PD_r_ as the reference signal. The transmitted beam is further split into two beams by a polarizing beam splitter (PBS), which are then diffracted by the reference and measurement gratings, respectively. The diffracted beams are then recombined at the PBS. After passing through a polarizer, the recombined beams interfere and are detected by PD_m_ as the measurement signal. The frequency of the reference signal remains constant, while the frequency of the measurement signal shifts due to the Doppler effect when the measurement grating moves.

The two light sources with frequencies $f_{1}$ and $f_{2}$ are expressed as

(8) $$E_{1}=A_{1}cos\left(2\pi f_{1}t+\phi_{01}\right)$$

(9) $$E_{2}=A_{2}cos\left(2\pi f_{2}t+\phi_{02}\right)$$

respectively, where $A_{1}$ and $A_{2}$ are the amplitudes, and $\phi_{01}$ and $\phi_{02}$ are the initial phases. Due to bandwidth limitations, a PD can only respond to low-frequency signals. The signals detected by PD_r_ and PD_m_ can be expressed as follows:

(10) $$I_{r}\propto A_{1}^{2}+A_{2}^{2}+2A_{1}A_{2}cos\left[2\pi \left(f_{1}-f_{2}\right)t+\phi_{r2}-\phi_{r1}\right]$$

(11) $$\begin{array}{cc} I_{m} & \propto A_{1}^{2}+A_{2}^{2}+2A_{1}A_{2}cos\left[2\pi \left(f_{2}-f_{1}+\Delta f_{k}\right)t+\phi_{m2}-\phi_{m1}\right] \end{array}$$

(12) $$\begin{array}{cc} \; & \propto A_{1}^{2}+A_{2}^{2}+2A_{1}A_{2}cos\left[2\pi \left(f_{2}-f_{1}\right)t+\Delta \phi_{k}+\phi_{m2}-\phi_{m1}\right] \end{array}$$

Here, $\phi_{r1}$, $\phi_{r2}$, $\phi_{m1}$, and $\phi_{m2}$ denote the phases of the reference and measurement diffracted beams, respectively. $\Delta f_{k}$ denotes the frequency shift induced by the Doppler effect during grating movement, and $\Delta \phi_{k}$ represents the phase variation converted from the frequency shift. The relationship between the measured grating displacement *L* and the phase variation ϕ is given by

(13) $$L=k\Delta \phi_{k}$$

where *k* is the proportionality coefficient, which is related to the grating parameters and the optical system structure.

The heterodyne laser interferometer also produces heterodyne signals, as illustrated in Figure 3b. Compared with the heterodyne grating interferometer, it replaces the measurement grating with a measurement mirror. When the measurement mirror moves, the reflected beam experiences a frequency shift due to the Doppler effect. The resulting heterodyne signal pattern is essentially identical to that of the grating interferometer and is described by Formulas (10) and (12).

### 2.3. Comparison of Homodyne Signals and Heterodyne Signals

In the field of optical interferometric metrology, homodyne and heterodyne interferometric signals differ significantly in their underlying principles, signal characteristics, and applications.

**Figure 4:**
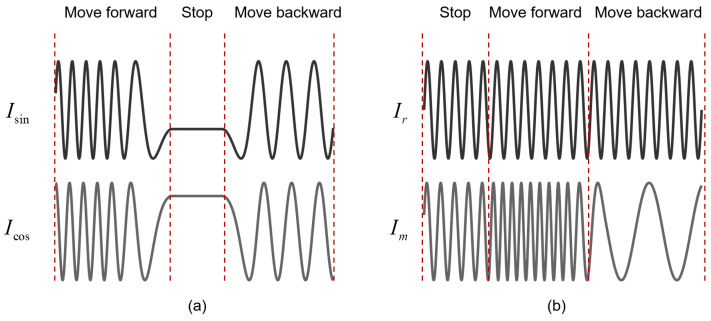
Comparison between homodyne signal and heterodyne signal. (**a**) Ideal homodyne signal. (**b**) Ideal heterodyne signal.

In terms of signal characteristics, homodyne interferometry utilizes a single-frequency laser source. The laser beam is split into a reference beam and a measurement beam by a beam splitter. Their interference at the detector generates a DC-modulated signal, whose intensity varies sinusoidally with the displacement in the measurement grating or mirror, as illustrated in Figure 4a. The phase change of the signal is directly proportional to the displacement. In contrast, heterodyne interferometry employs a dual-frequency laser source, for example, a dual-frequency helium–neon laser, that emits two orthogonally linearly polarized beams at frequencies $f_{1}$ and $f_{2}$. The interference of these beams produces an alternating-current (AC) beat signal at a frequency of $|f_{1}-f_{2}|$. When the measurement grating or mirror moves, the Doppler effect induces a frequency shift in the measurement beam, causing a corresponding phase shift in the beat-frequency signal, as shown in Figure 4b. The displacement is then determined by measuring this phase change. Compared with heterodyne detection, homodyne signal processing is relatively simple but susceptible to DC drift. In contrast, the AC signal generated in heterodyne interferometry is better suited for processing with AC amplification circuits and more effectively suppresses DC-related noise.

Regarding noise immunity, homodyne interferometric systems are highly sensitive to environmental disturbances. Due to their DC-modulated signals, fluctuations in laser power, variations in detector dark current, and ambient stray light can cause baseline drift, thereby compromising measurement accuracy. For example, temperature-induced fluctuations in the air’s refractive index can alter the optical path length, introducing measurement errors. Mechanical vibrations may also cause jitter in the interference fringes, further complicating phase demodulation. In contrast, heterodyne interferometric systems offer significant advantages in noise immunity. The AC beat signal allows DC noise to be effectively suppressed using AC-coupled circuits, making the system largely immune to low-frequency disturbances such as laser power instability and stray light. Furthermore, the dual-frequency nature of heterodyne interferometry provides inherent common-mode rejection of environmental changes. For instance, temperature variations affect both frequency components similarly, leaving the beat frequency and phase largely unchanged. As a result, heterodyne interferometry demonstrates greater stability in complex environments such as industrial settings.

Measurement precision is a critical metric for evaluating interferometric system performance. Under ideal conditions, homodyne interferometry can achieve high precision through fringe subdivision techniques, theoretically reaching nanometer- or even sub-nanometer-scale resolution. However, in practical applications, its precision is often limited by sensitivity to environmental disturbances. For example, humidity or temperature fluctuations can introduce errors in high-precision displacement measurements. Heterodyne interferometry, by virtue of its unique signal characteristics and robust noise immunity, typically achieves higher measurement precision and stability. The phase-to-displacement relationship in heterodyne signals is more linear, and AC signal demodulation techniques such as lock-in amplification enable high-precision phase detection even in noisy environments. Furthermore, increasing the beat signal frequency (e.g., to tens of megahertz) enhances measurement resolution. When paired with high-precision counters, this allows for sub-nanometer-scale or even picometer-scale displacement measurements.

From the perspective of system complexity and cost, homodyne interferometric systems are relatively simple in structure, requiring only a single-frequency laser source and basic beam-splitting and -reflecting components. Consequently, they are cost-effective and suitable for cost-sensitive laboratory measurement scenarios with relatively stable environments. Heterodyne interferometric systems, however, require dual-frequency laser sources and more complex optical paths and signal processing circuits, resulting in higher system costs. Nonetheless, their performance advantages make them the preferred choice for high-precision measurements in harsh environments.

## 3. Optical Interferometric Signal Processing Techniques

### 3.1. Phase Estimation Methods for Homodyne Signals

**Figure 5:**
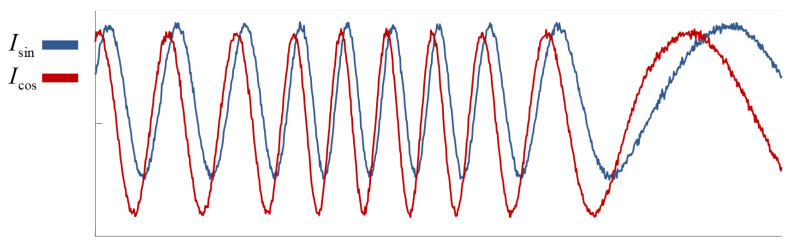
Homodyne signal with non-ideal components.

The displacement information is embedded in the phase of the homodyne signal, so the homodyne signal phase calculation accuracy directly determines the displacement measurement accuracy of the optical system. The ideal homodyne signal is expressed as Formulas (6) and (7). However, in practical homodyne optical systems, due to factors such as assembly deviations and nonlinear errors of optical components, the detected signals usually contain DC drift, amplitude drift, phase error, and high-frequency noise, as shown in Figure 5.

An actual homodyne signal with non-ideal components can be expressed as

(14) $$I_{cos}\left(t,L\right)=A_{1}\left(t,L\right)cos\left(\Delta \phi_{m}\right)+D_{1}\left(t,L\right)+n_{1}\left(t,L\right)$$

(15) $$I_{sin}\left(t,L\right)=A_{2}\left(t,L\right)sin\left(\Delta \phi_{m}+\phi_{2}\left(t,L\right)\right)+D_{2}\left(t,L\right)+n_{2}\left(t,L\right)$$

where $A_{1}\left(t,L\right)$ and $A_{2}\left(t,L\right)$ are the amplitudes of the two homodyne signals, which vary with time and the displacement in the measurement target; $\phi_{2}\left(t,L\right)$ is the phase error between the two signals, which destroys the orthogonality of the homodyne signals; $D_{1}\left(t,L\right)$ and $D_{2}\left(t,L\right)$ are the DC components of the signals, which also change with time and the displacement in the measurement target; and $n_{1}\left(t,L\right)$ and $n_{2}\left(t,L\right)$ are high-frequency noise components.

These errors introduce significant deviations into phase estimation, severely affecting the displacement measurement accuracy of the optical system. Therefore, these error factors must be considered when calculating the phase of homodyne signals. Currently, the calculation methods for homodyne signals are mainly divided into two categories. The first comprises the error calibration methods, which design a corresponding correction algorithm for each type of error, progressively correct the four main errors mentioned above, finally obtain sine–cosine signals close to the ideal state, and then calculate the phase through the arctangent function or other phase linearization functions. The second includes the ellipse parameter estimation methods, which use the two signals as the x-axis and y-axis coordinates to construct a Lissajous figure. Non-ideal errors cause the Lissajous figure to take an elliptical shape, and the signal phase can be obtained by estimating the ellipse parameters.

#### 3.1.1. Error Calibration Methods

**Figure 6:**
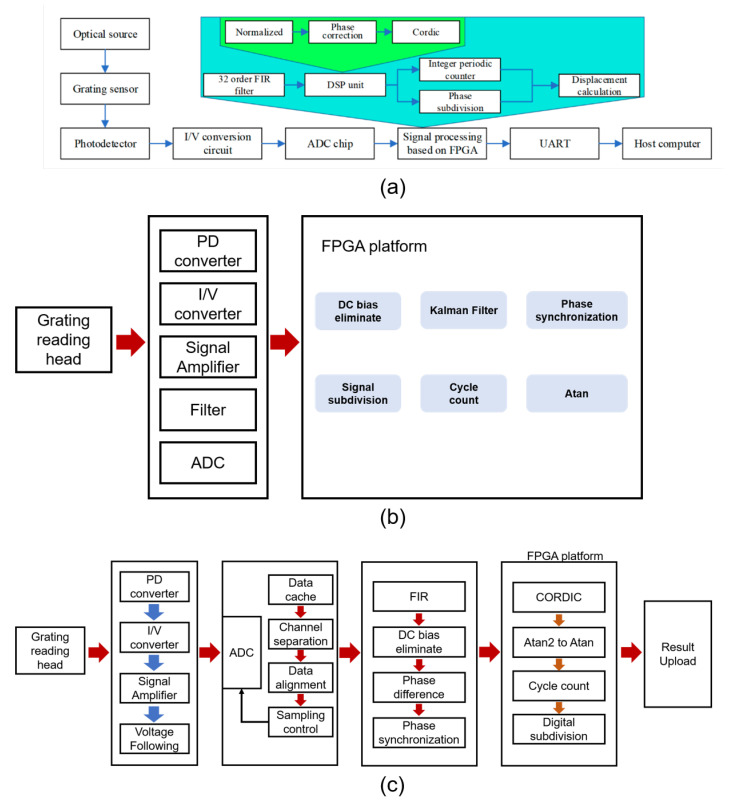
Examples of error calibration methods. (**a**) Method based on FIR filter and phase correction [133]. (**b**) Method based on Kalman filter [136]. (**c**) Method based on phase synchronization [135].

Error correction methods represent a straightforward approach to suppressing noise in homodyne signals. Y. Han first digitized homodyne signals by using an ADC and performed subsequent processing with an FPGA [133], as shown in Figure 6a. Then, FIR filters were applied to filter the signals, removing high-frequency noise and DC components. Next, signal normalization was conducted by acquiring one period of the signal, calculating its maximum value, and dividing all signal points in that period by this maximum. Subsequently, a pulse-counting method was used to measure and correct the phase difference between the two signals, restoring their orthogonality. After these correction steps, two sinusoidal signals free of DC components were obtained, from which the phase was calculated using the arctangent operation. Although this method effectively mitigates the impact of non-ideal components on phase calculation, it requires substantial computational resources and introduces high latency, primarily due to the division operations required for each signal point during the normalization step. N. Shi adopted a similar signal correction approach [136], as shown in Figure 6c. Building on their work, Y. Wang replaced the FIR filter with a Kalman filter [135], as shown in Figure 6b. By leveraging the sinusoidal characteristics of the input signals, the Kalman filter achieved high-frequency noise suppression, signal normalization, and phase correction at the same time with lower computational complexity. This not only significantly reduced computational resource consumption but also minimized the computational delay to 1.26 us, greatly enhancing the real-time performance of the calculation.

#### 3.1.2. Ellipse Parameter Estimation Methods

**Figure 7:**
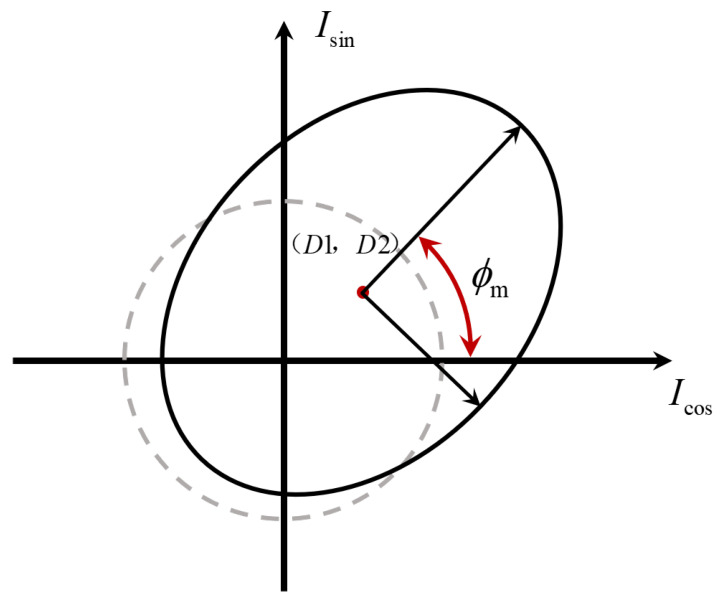
Lissajous ellipse for ellipse parameter estimation.

In homodyne grating and laser interferometry, ellipse parameter estimation methods demonstrate unique advantages in phase retrieval by modeling the Lissajous figure as an ellipse [51,137,138,139,140,141,142,143,144,195]. These approaches take the two orthogonal signals generated by a homodyne interference system as the horizontal and vertical coordinates of an ellipse and retrieve phase information by fitting geometric parameters such as major axis, minor axis, and rotation angle, as shown in Figure 7. Conventional methods for elliptic parameter estimation primarily include the least squares method [137,140] and the Kalman filtering method [196]. In homodyne grating interference systems, non-ideal factors arising from assembly errors and nonlinearities in optical components—including DC drift, amplitude imbalance, and phase error—cause the signal trajectory to deviate from an ideal circle. Ellipse parameter estimation methods mitigate these errors through least squares fitting or nonlinear optimization algorithms.

Based on Equations (14) and (15), after filtering out high-frequency noise, the phase information of the homodyne signal can be expressed as

(16) $$\Delta \phi_{m}=\frac{L}{k}=arctan\left(\frac{I_{sin}-D2}{\left(I_{cos}-D1\right)\alpha +\left(I_{sin}-D2\right)\beta }\right)$$

where the correction coefficients α and β can be represented as

(17) $$\alpha =\frac{A_{2}}{A_{1}cos\left(\phi_{2}\right)}$$

(18) $$\beta =tan(\phi_{2})$$

The equation of the ellipse is given by

(19) $$AI_{cos}^{2}+BI_{cos}I_{sin}+CI_{sin}^{2}+DI_{cos}+EI_{sin}+F=0$$

The DC components $D_{1}$ and $D_{2}$ of the signal can be expressed in terms of the parameters in the ellipse equation as

(20) $$D_{1}=\frac{2CD-BE}{B^{2}-4AC}$$

(21) $$D_{2}=\frac{2AE-BD}{B^{2}-4AC}$$

The correction coefficients α and β can be represented using the parameters of the ellipse equation as

(22) $$\alpha =\frac{2A}{\sqrt{4AC-B^{2}}}$$

(23) $$\beta =\frac{B}{\sqrt{4AC-B^{2}}}$$

Based on Equations (16)–(23), a relationship can be established between the phase information of the homodyne signal and the ellipse parameters A, B, C, D, E, and F. Thus, by accurately estimating these elliptic parameters, the phase information of the homodyne signal can be derived.

Table 1 shows the comparison between error calibration methods and ellipse parameter estimation methods. Although the error calibration methods can achieve high resolution, their step-by-step calibration consumes substantial hardware computing resources, leading to increased costs. As a result, they are commonly applied in academia. The ellipse parameter estimation methods are more flexible in their implementation: they can utilize Kalman filtering to achieve extremely high precision or employ the least squares method for the pipelined processing of signals. With lower hardware resource consumption, they are widely used in industry.

### 3.2. Phase Estimation Methods for Heterodyne Signals

Phase calculation for heterodyne interferometers represents one of the key steps towards achieving high-precision displacement measurement, with its essence lying in converting optical frequency shift signals into phase information and realizing high-resolution demodulation. Depending on distinct signal processing principles, existing approaches are primarily categorized into three types: pulse-counting methods [5,197,198,199,200,201,202,203,204,205,206], quadrature phase-locked methods [146,147,148,149,150,207,208], and the Kalman filtering method [151]. In pulse-counting methods, after heterodyne interference signals are converted into sine waves by the PD, a comparator is required to transform them into square waves. The latter two method types, conversely, utilize an analog-to-digital converter (ADC) to acquire sine waves for direct amplitude–phase analysis.

Pulse-counting methods achieve phase calculation through high-frequency pulse filling and time difference measurement, relying on GHz level phase-locked loop (PLL) frequency multiplication technology to enable sub-nanometer resolution and high-speed measurement capabilities. Quadrature phase-locked methods extract phase information via quadrature demodulation and arctangent operation, demonstrating remarkable advantages in picometer-scale resolution. The Spectrum Analysis Method performs phase analysis in the frequency domain, allowing for the easier separation of the signal fundamental frequency from harmonic interferences and exhibiting strong anti-harmonic capabilities. The subsequent discussion systematically addresses the principles, evolutionary, and technological breakthroughs of these three method types.

#### 3.2.1. Pulse-Counting Methods

Pulse-counting methods obtain phase information by using an additional high-frequency clock signal to count the periods of input measurement signals.

These methods convert measurement and reference signals into square waves via a zero-crossing comparator and then input these square waves into counters. By calculating the integer number of signal periods and the number of reference signal periods corresponding to the sub-period segment, the phase difference between the two signals can be accurately determined [197,198].

To improve the resolution of phase measurement, a common approach is to apply PLL-based frequency multiplication to the interference signal, thereby increasing the phase subdivision factor. However, due to the bandwidth limitations of logic devices, the multiplication factor achievable by PLL-based techniques is constrained. To address this issue, Hu proposed a phase measurement method based on PLL frequency multiplication combined with digital delay lines [209]. On the basis of PLL-based multiplication, this method introduces multistage digital delay lines to further enhance the effective phase subdivision factor, thereby improving the resolution of phase and displacement measurements. A series of interferometric signal processing boards have been developed based on this method, achieving an electronic subdivision factor of up to 1024. This enables a maximum measurement resolution of 0.31 nm @optical 2-fold subdivision or 0.15 nm@optical 4-fold subdivision and a maximum measurement speed of 2 m/s@optical 2-fold subdivision or 1 m/s@optical 4-fold subdivision [209,210]. Frank et al. also implemented a heterodyne interferometric phase demodulation system based on this method, achieving a measurement resolution of 0.31 nm and a measurement speed of 2.1 m/s [5].

**Figure 8:**
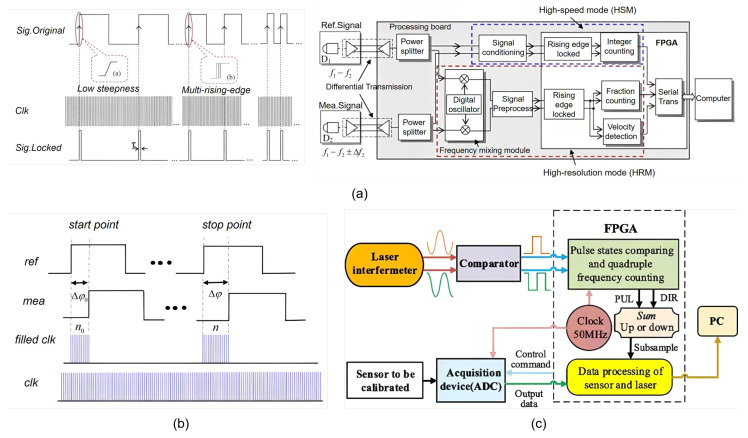
Examples of pulse-counting methods. (**a**) Method based on rising-edge locking with high-frequency clock signals and digital frequency mixing [170]. (**b**) Method for movement direction identification and phase correction [145]. (**c**) Method based on fringe-to-pulse counting [171].

E. Zhang proposed a novel signal processing method for laser heterodyne interferometry based on rising-edge locking with high-frequency clock signals and digital frequency mixing [170], as shown in Figure 8a. By locking the rising edges of reference and measurement signals with a high-frequency clock and replacing traditional analog mixing with digital frequency mixing, it eliminates multi-edge counting errors and enhances anti-interference capability. Experiments verify that it achieves both high speed, 2.4 m/s, and resolution, 0.008 nm, providing a new solution for high-precision displacement measurement.

T. Yang proposed a novel signal processing method for movement direction identification and phase correction in laser heterodyne interferometry [145], as shown in Figure 8b. The core approach involves establishing four 90° phase intervals based on the reference signal, realizing real-time direction identification and integer fringe counting by detecting the times the rising edge of the measurement signal crosses these intervals. A detailed phase correction method was proposed to solve the fractional phase compensation issue when the initial phase difference is non-zero. The innovation lies in integrating direction identification with phase correction, determining the movement direction through interval division and state changes of the measurement signal, which overcomes measurement errors caused by initial phase differences in traditional methods. Experiments verify the method’s feasibility in bidirectional movement stability tests and nano/micrometer displacement measurements, achieving a measurement accuracy of 6.84 nm. This provides an effective solution for high-precision displacement measurement.

J. Zhou proposed a novel laser interferometric demodulation method based on fringe-to-pulse counting, as shown in Figure 8c, which converts high-frequency orthogonal analog signals into digital signals, obtains displacement increments by comparing four states with a high-frequency clock, and reduces the data rate via subsampling [171]. The innovation lies in eliminating the need for high-speed data acquisition devices with simple hardware and algorithms, enabling real-time high-precision demodulation for both low- and medium-frequency vibration calibrations. Experiments verify that amplitude and phase deviations are within allowable limits.

Pulse-counting methods are widely applied in practice due to their simple algorithm and ease of implementation. They also feature strong algorithm robustness, high sensitivity, and suitability for dynamic measurement tasks. Currently, commercial boards based on pulse-counting methods are available globally. For instance, Agilent has released the N1225A board with 0.15 nm resolution [211]; ZYGO offers the ZMI4104(C) board, also with 0.15 nm resolution [212]. However, inherent limitations exist. First, their accuracy is largely constrained by the precision of zero-crossing conversion, with errors increasing significantly in low-SNR environments. Second, resolution and precision degrade gradually as the frequency of the measured signal increases. Finally, high-frequency signals after multiplication require advanced chip performance.

#### 3.2.2. Quadrature Phase-Locked Methods

Quadrature phase-locked methods extract signal amplitude and phase information through digital signal processing techniques such as mixing and filtering, breaking through the limitation of pulse-counting methods, which rely on periodic counting [213,214,215,216,217].

At present, most mainstream quadrature phase-locked loop techniques employ digital circuits to perform critical operations such as signal mixing and filtering. In comparison with conventional lock-in amplifiers implemented using analog circuits, the digital quadrature phase-locked loop features high precision, free from errors caused by the uncertainty of analog circuits; meanwhile, the algorithm demonstrates better stability, maintaining stable measurement performance over long periods. Additionally, it offers good flexibility, allowing for convenient modification of the algorithm form according to different measurement requirements, thus solving many difficulties faced by analog phase-locked circuits during upgrading [218].

**Figure 9:**
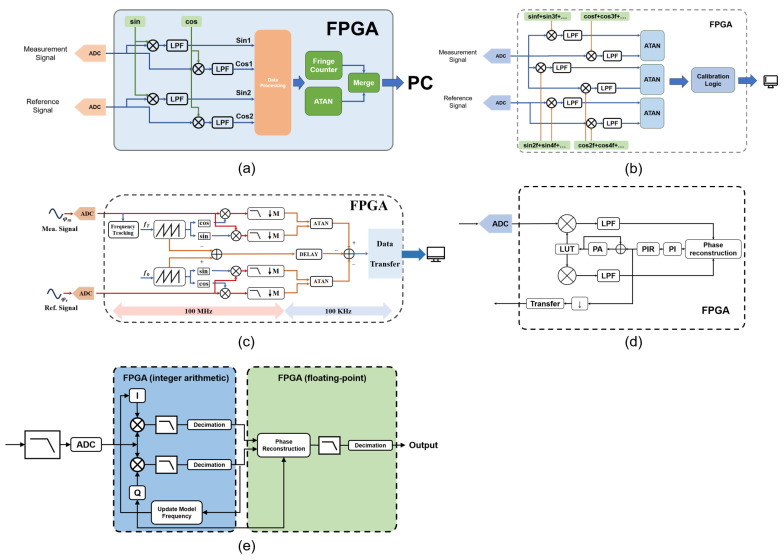
Examples of quadrature phase-locked methods. (**a**) Method using digital lock-in phase principle based on FPGA and high-speed ADC [146]. (**b**) Method based on digital dual-frequency comb [147]. (**c**) Enhanced lock-in method based on pulse counting for frequency tracking [150]. (**d**) Method with phasemeter scheme based on the DPLL [148]. (**e**) Method with quadrature phase-locked phase detection scheme based on the DPLL [149].

P. K. proposed a lock-in phase measurement method based on an FPGA and a high-speed ADC, which extracts phases of heterodyne interferometric signals using the digital lock-in principle and achieves picometer-level resolution through dynamic window filtering and Doppler frequency shift compensation [146], as shown in Figure 9a. It uses a commercial ADC board with user-defined algorithms to simultaneously track dual-beat frequencies, overcoming bandwidth limitations of traditional phasemeters. Experiments demonstrate a long-term stability of 0.8 pm and reveal polarization effects as the primary error source.

P. Hu proposed a novel phase measurement method based on a digital dual-frequency comb, which forms bandpass filter banks using intrinsic frequency comb signals and narrowband low-pass filters to divide broadband signals from high-speed motion into multiple narrow bands for SNR improvement [147], as shown in Figure 9b. It simultaneously uses two groups of comb signals for phase measurement and output rotation. It solves phase measurement inaccuracies caused by the input signal frequency being the average of two comb frequencies during high-speed motion, enabling high-speed and high-precision phase measurement for heterodyne interferometry.

S. Lu proposed an enhanced lock-in method based on pulse counting for frequency tracking which roughly estimates the measurement signal frequency via pulse counting and generates orthogonal mixing signals to achieve dynamic frequency tracking on an FPGA platform [150], as shown in Figure 9c. The innovation lies in addressing the sensitivity to filter performance and poor generalization capability of traditional quadrature phase-locked methods, achieving resolution errors within tens of picometers and a measurement speed exceeding 1 m/s, thereby significantly enhancing the robustness of the lock-in method.

Taiji used a phasemeter scheme based on the digital phase-locked loop (DPLL), implementing signal mixing, filtering, and frequency tracking via an FPGA and adopting common-mode noise rejection to reduce sampling and frequency jitter noises [148], as shown in Figure 9d. It achieves a sensitivity of $2\pi \mu rad/\sqrt{Hz}$, meeting the requirements for 0.01 Hz–1 Hz and 0.1 mHz–1 mHz frequency bands, thus providing key technical support for the Taiji space gravitational wave detection mission.

The phasemeter designed for LISA employs a quadrature phase-locked phase detection scheme based on the DPLL [149], as shown in Figure 9e. To achieve phase locking between the oscillator and the input signal, the scheme utilizes residual tracking error information from two mixed signals to feedback-correct the phase of the digital oscillating signal. Meanwhile, data acquired by the ADC are transmitted to the phase-locked output module to lock the laser for stable acquisition of the required heterodyne frequency. This approach achieves a phase measurement accuracy of $6\pi \mu rad/\sqrt{Hz}$ in the 1 mHz–1 Hz frequency band.

The advantages of quadrature phase-locked methods are mainly reflected in the following aspects: first, they make full use of signal amplitude information, which can more effectively utilize the information carried by the signal compared with pulse-counting methods, thus improving measurement accuracy; second, they have strong noise resistance, maintaining good measurement performance in noisy environments; third, they can achieve higher precision and resolution to meet the needs of high-precision measurements. However, the selection of the cutoff frequency of the low-pass filter in these methods has an important impact on the dynamic range and output signal noise, which need to be adjusted according to specific measurement scenarios and requirements in practical applications.

#### 3.2.3. Kalman Filtering Method

In heterodyne signal demodulation, quadrature phase-locked methods extract phase information through mixing and filtering, but they exhibit sensitivity to filter performance and tend to suffer from accuracy degradation due to fixed parameters in dynamic or high-noise scenarios. They also struggle to balance real-time performance and accuracy. In contrast, Kalman filtering employs state-space modeling, treating phase, frequency, and amplitude as state variables, and dynamically updates estimations via a prediction-correction mechanism, effectively suppressing random noise and nonlinear interference [219,220,221,222,223]. Its advantages include (1) the adaptive tracking of signal variations without fixed filter parameters; (2) low-latency computation relying only on current sampling data, suitable for real-time scenarios; and (3) enhanced robustness against dynamic frequency drift and environmental noise, making it particularly applicable to high-precision dynamic measurements such as multi-degree-of-freedom grating interferometry.

**Figure 10:**
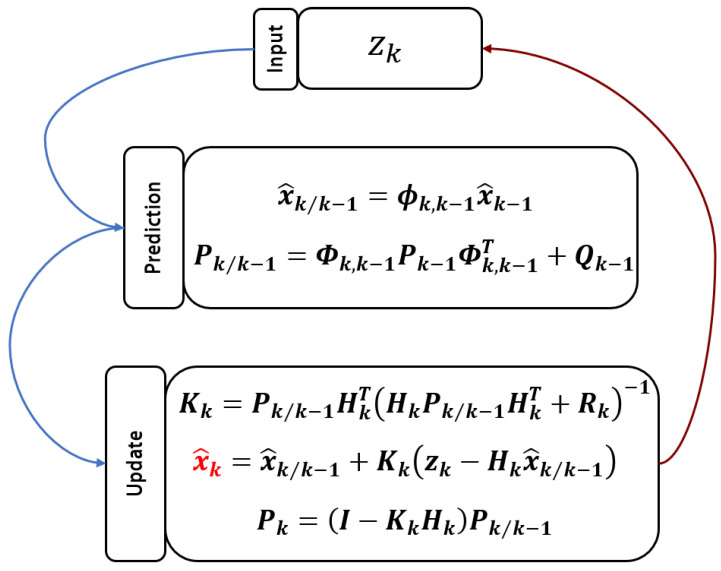
Calculation process of Kalman filter [151].

Y. Ma proposed a heterodyne signal phase solution algorithm based on the first-order extended Kalman filter (EKF), which sets the phase, frequency, and amplitude of the sinusoidal signal as state variables and implements prediction-correction computation via an FPGA [151]. The processing framework of the extended Kalman filter with an FPGA shows in Figure 10. The advantages include (1) achieving a measurement accuracy of 0.03° and a resolution of 0.01°, (2) enabling low-latency computation suitable for high-speed dynamic scenarios, and (3) featuring compact hardware deployment to enhance the real-time performance and noise immunity of multi-degree-of-freedom grating interferometers.

Table 2 shows a comparison of pulse-counting methods, quadrature phase-locked methods, and the Kalman filtering method. Pulse-counting methods exhibit low signal demodulation resolution due to signal zero-crossing errors and clock frequency limitations. However, they do not rely on high-speed ADCs, resulting in lower costs, and thus find applications in both academia and industry. Quadrature phase-locked methods can achieve higher resolution but rely on high-speed ADCs, leading to higher costs. Owing to their excellent performance, they have extensive applications in both academia and industry. The Kalman filtering method can achieve higher resolution than quadrature phase-locked methods. Nevertheless, due to its algorithmic constraints, it cannot implement pipelined signal processing, which limits its signal processing bandwidth. As a result of this limitation, it is generally only applied in academia.

## 4. Electronic Platforms for Signal Processing

**Figure 11:**
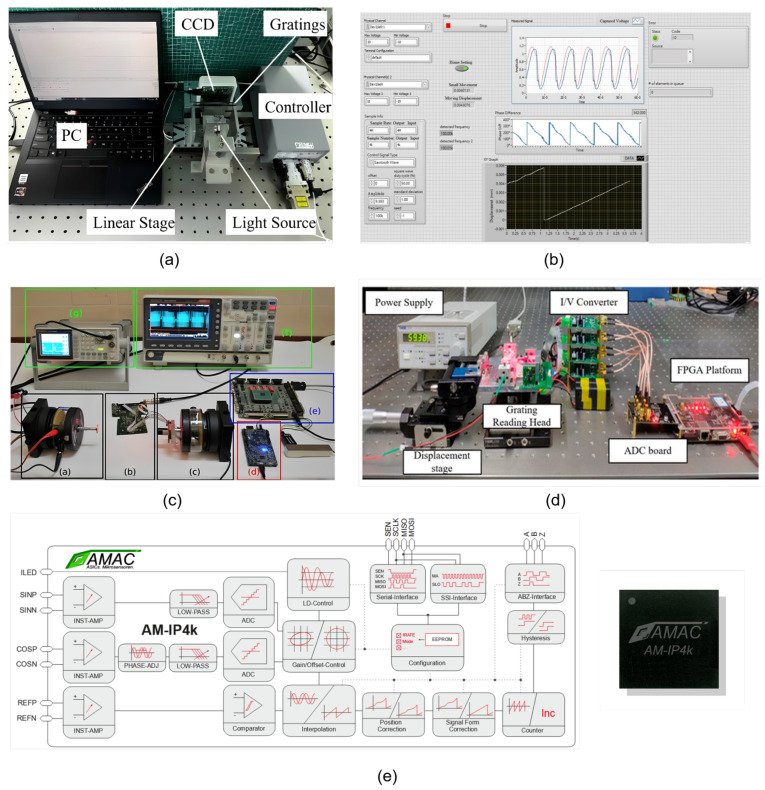
Hardware platforms for optic interference signal processing. (**a**) PC platform [157]. (**b**) Labview interface on PC. (**c**) MCU/DSP platform [162]. (**d**) FPGA platform [133]. (**e**) ASIC platform [224].

As a core technology in precision displacement metrology, optical interferometry imposes diverse requirements on hardware platforms for its signal processing, encompassing both homodyne and heterodyne signals, with respect to computational capacity, real-time performance, power consumption, and cost. Current mainstream hardware platforms can be categorized into four major types—personal computers (PCs) (Figure 11a,b), microcontrollers/digital signal processors (MCUs/DSPs) (Figure 11c), field-programmable gate arrays (FPGAs) (Figure 11d), and application-specific integrated circuits (ASICs) (Figure 11e)—each suited to full lifecycle scenarios ranging from algorithm verification to industrial mass production. PCs, relying on robust general-purpose computational capabilities and rich software ecosystems, serve as the primary choice for complex algorithm development in laboratory environments. MCUs/DSPs, leveraging their low-cost and low-power advantages, dominate simple signal processing in miniaturized and portable sensors. FPGAs, through hardware logic parallelism, achieve high real-time performance requirements and are well-suited for high-speed feedback control in industrial settings. ASICs, with their ultra-high performance and integration, support large-scale production of high-end grating sensors. These four kinds of platforms complement each other in terms of computational complexity, latency, flexibility, and cost, collectively forming the hardware technical framework for optical interferometric signal processing.

### 4.1. PCs

PCs represent the most versatile hardware platform for grating interferometric signal processing, with their core value lying in providing a flexible software environment for algorithm development and verification. PCs are built on general-purpose processors based on x86 or ARM architectures, equipped with large-capacity memory and high-speed storage, and support multi-task parallel processing. At the software level, PCs can run professional tools such as MATLAB [225,226,227], Python (paired with NumPy/SciPy toolkits) [228,229,230], and LabVIEW [231,232,233,234,235,236,237,238,239], enabling the rapid implementation of complex algorithms. Their computational capacity is typically measured in GFLOPS (gigaflops, billions of floating-point operations per second), with modern PCs achieving a single-threaded computational capacity of 10 to 100 GFLOPS and exceeding 1 TFLOP (teraflop, one trillion floating-point operations per second) under multi-threaded parallel processing.

PCs are primarily utilized for algorithm prototyping and performance validation in laboratory environments [152,153,154,155,156,157]. For homodyne interferometric signals, phase demodulation requires suppressing DC drift, and PCs can achieve sub-nanometer phase measurement accuracy through complex algorithms such as digital filtering and elliptical fitting to compensate for non-orthogonal errors [233]. For heterodyne interferometric signals, their beat-frequency signals require frequency tracking and phase subdivision [240]. PCs can leverage FFT to analyze beat-frequency stability or employ PLL algorithms to realize high-precision demodulation of dynamic displacement. Furthermore, PCs support joint analysis of multi-signals, providing technical support for system-level error modeling.

The core advantages of PCs lie in their development flexibility and complex task handling capability. Their software ecosystems support rapid algorithm iteration such as adjusting filtering parameters and optimizing fitting models, without requiring hardware redesign; simultaneously, they enable synchronized signal acquisition via DAQ cards, processing, display, and storage, significantly facilitating debugging and analysis. However, the limitations of PCs are also pronounced: their size and power consumption fall short of the requirements for miniaturized devices; the millisecond-level task scheduling latency of general-purpose operating systems cannot accommodate real-time feedback demands in industrial settings, which have microsecond-level latency scenarios in motion control; and the cost of high-performance PCs typically reaches several thousands dollars, making them unsuitable for large-scale deployment.

### 4.2. MCUs/DSPs

MCUs and DSPs, serving as the core components of embedded systems, exhibit a clear functional division in grating interferometric signal processing: MCUs primarily handle low-complexity control and simple signal processing tasks, while DSPs are dedicated to the efficient implementation of digital signal processing algorithms.

MCUs integrate peripherals such as CPUs, RAM, Flash, ADCs/DACs, etc., with a computing capability of approximately 0.1 to 10 DMIPS (Dhrystone million instructions per second) and power consumption as low as 1 to 100 mW. Their abundant on-chip resources are suitable for implementing logic control and simple signal processing such as low-pass filtering to suppress high-frequency noise and threshold detection for identifying interference fringe zero-crossings. A typical application scenario is low-cost displacement sensors [162,241], where interferometric signals are acquired via on-chip ADC and rough displacement values are output after simple mean filtering.

DSPs are optimized for digital signal processing, featuring built-in hardware multipliers, pipeline architectures, and parallel arithmetic units. They achieve computing capabilities of 100 to 1000 DMIPS with power consumption ranging from 100 mW to 2 W. Their instruction sets are optimized for FFT, convolution, and correlation operations, making them suitable for the real-time processing of high-frequency signals [242]. For example, quadrature demodulation of heterodyne interference that converts MHz level beat-frequency signals into I/Q baseband signals and phase subdivision of homodyne signals that subdivides fringe periods into 1/1000 via arctangent operations can both achieve microsecond-level demodulation through DSP hardware acceleration units.

The core advantages of MCUs/DSPs lie in their low cost and low power consumption [243,244,245]. MCUs are priced at only 0.1 to 10 USD, while DSPs cost approximately 10 to 100 USD, making them suitable for low-cost sensors in mass production. Their power consumption supports battery-powered portable devices. Furthermore, on-chip peripherals such as ADCs and PWMs reduce external circuitry, simplifying system design. However, both exhibit limited computational capabilities: MCUs cannot handle complex algorithms such as high-order filtering and multi-parameter fitting, and DSPs are prone to computational resource saturation under high sampling rates or multi-channel signals. Meanwhile, modifying the code after programming requires recompilation and download, hindering rapid algorithm iteration.

### 4.3. FPGAs

FPGAs realize signal processing through configurable hardware logic gates, serving as the optimal balance platform for real-time performance and flexibility in grating interferometric signal processing. Based on SRAM or Flash process technologies, their programmable logic units and flip-flops support hardware parallel computing and pipeline design. Mainstream FPGAs such as Xilinx Kintex and Intel Stratix contain tens of thousands to millions of logic elements, integrating DSP slices, high-speed ADC interfaces, and high-speed serial transceivers. They achieve computational latency at the nanosecond level and throughput at the GHz level while supporting dynamic reconfiguration.

The core advantage of FPGAs lies in hardware-level real-time performance, making them particularly suitable for scenarios such as high-speed signal acquisition and preprocessing, as well as real-time phase subdivision [246,247,248,249]. For instance, the beat-frequency signal of heterodyne interference requires sampling at rates above 50 MHz. FPGAs can achieve delay-free sampling via high-speed ADC interfaces and complete preprocessing steps like digital down-conversion and low-pass filtering in hardware pipelines, eliminating the software latency of PC platforms [133]. For phase subdivision in homodyne interference, FPGAs can implement high-precision arctangent operations through customized logic units, subdividing the interference fringe period into 1/1000 to meet the measurement requirements of ultra-precision equipment such as lithography machines [134,135,136]. Furthermore, FPGAs support multi-channel parallel processing, making them suitable for multi-dimensional displacement measurement systems.

FPGAs have excellent real-time performance and flexible configurability. Their hardware parallel architecture enables nanosecond-level latency, meeting the real-time feedback requirements of industrial sites. The dynamic reconfiguration function supports online adjustment of algorithm parameters to adapt to different measurement scenarios. However, FPGAs have a high development threshold, requiring mastery of hardware description languages like Verilog/VHDL, and their design cycles span months [250]. With chip costs ranging from hundreds to thousands of dollars and power consumption from several to tens of watts, FPGAs are more expensive and power-hungry than MCUs/DSPs, limiting their application in low-cost scenarios.

### 4.4. ASICs

ASICs are custom-designed integrated circuits tailored for specific functions, achieving ultimate optimization in performance and integration for optical interferometric signal processing through hardware-embedded specific processing logic, such as AM-IP4k [224] and GC-NIP for homodyne signals. Based on CMOS technology, ASICs integrate signal acquisition, processing, and output into a single chip, with an area typically at the square-millimeter scale, power consumption as low as the microwatt level, and processing speeds reaching the GHz range.

The core value of ASICs lies in their scale advantages in performance and cost, making them suitable for high-end grating sensors in mass production. Take the displacement measurement module of lithography machines as an example: it needs to simultaneously meet the requirements of sub-nanometer precision, microsecond-level latency, and extremely small size. Furthermore, the mass production cost of ASICs decreases significantly with the increase in yield—when produced in million-unit quantities, the cost per chip can be as low as a few dollars, making them ideal for large-scale deployment of industrial-grade sensors.

ASICs have ultimate performance and ultra-low power consumption. Their customized hardware logic avoids the redundant calculations of general-purpose platforms, enabling processing latency as low as the nanosecond level and power consumption only 1/10 to 1/100 of that of FPGAs. The high integration (full functionality on a single chip) significantly simplifies system design. However, ASICs entail extremely high development costs and extremely long development cycles. Once the design is finalized, modifications are difficult, making ASICs suitable only for scenarios with clear requirements and large production volumes.

Table 3 shows a comparison of different hardware platforms for interferometer signal processing. Among all hardware platforms, PCs exhibit the highest flexibility. Developers can rapidly develop and validate new algorithms on PCs. However, their power consumption, size, and parallel processing capability have hindered large-scale industrial applications, limiting their use to academic research platforms. FPGAs and DSPs strike a balance among flexibility, size, and power consumption. While their flexibility is inferior to that of PCs, they feature a smaller size and lower power consumption, thus finding extensive applications in industry. Furthermore, FPGAs, due to their parallel computing capability, are often employed in multi-channel, high-bandwidth signal processing. ASICs exhibit the lowest flexibility, with long development cycles and high upfront development costs. Nevertheless, they offer low deployment costs, along with minimal size and power consumption, which makes them commonly used in large-scale industrial applications, typically combined with ellipse parameter estimation methods.

## 5. Conclusions

This paper reviews recent developments in signal processing techniques and platforms for optical interferometric measurement, focusing on both homodyne and heterodyne systems. The goal of signal processing is to enable high-precision, multi-degree-of-freedom, and large-range displacement measurement from the perspective of signal processing.

As one of the leading non-contact methods for precision displacement measurement, optical interferometry offers significant advantages in terms of accuracy, speed, and reliability. It has been widely applied in various industrial sectors, including mechanical engineering, electronics manufacturing, and aerospace. While novel interferometric architectures continue to emerge, increasing attention has been drawn to the importance of signal conditioning techniques for system induced errors. Algorithms that offer high precision, low latency, and minimal computational load have become essential to enabling the deployment of optical interferometers in real-world applications.

Homodyne interferometry employs a single-frequency laser and derives displacement by analyzing phase variations in a DC amplitude-modulated signal. This method features a relatively simple system design but is sensitive to laser stability and environmental noise and is prone to intensity fluctuations and DC drift. Signal processing for homodyne systems primarily focuses on noise suppression, normalization and orthogonalization of the dual sinusoidal signals, and fine phase subdivision. Ellipse parameter estimation methods calibrate orthogonal signals by analyzing their Lissajous figures, from which phase information is extracted. Error calibration methods align the phase of orthogonal signals using known phase relationships.

Heterodyne interferometry utilizes a dual-frequency laser to generate a dynamic beat-frequency signal. Displacement is determined by demodulating phase or frequency variations in this modulated signal. By incorporating frequency-shifting techniques, heterodyne systems achieve effective separation of signal and noise, significantly enhancing the signal-to-noise ratio and robustness against interference. These systems typically offer a broader dynamic range and higher linearity. Signal processing methods for heterodyne signals are more diverse. Pulse-counting methods are computationally simple but are limited by signal quality, frequency stability, and their reliance on periodic counting. Quadrature phase-locked methods, which employ digital phase-locked loop techniques and leverages signal amplitude information, perform better than pulse counting in low-SNR environments. The Kalman filtering approach estimates phase parameters in real time and is well-suited for dynamic applications due to its responsiveness.

As lithography technology advances toward ever-smaller process nodes, the need for the precise, real-time, multi-degree-of-freedom positioning of stages and masks in lithographic systems is becoming increasingly critical. Future development of signal conditioning algorithms will need to address not only the errors arising from optical component imperfections (such as virtual reflections at optical interfaces and non-ideal behaviors of optoelectronic sensors) but also the coupling effects between different degrees of freedom and the influence of these couplings on measurement accuracy. At the same time, reconciling the trade-offs among large-range measurement, high-speed target tracking, and sub-nanometer precision will be a key direction in the evolution of interferometric signal processing techniques. Additionally, the rise of machine learning has opened up new breakthrough directions for interferometric signal processing. Machine learning methods enable more precise analysis and correction of demodulation error patterns while also allowing for the incorporation of information such as ambient temperature and humidity into the model. Furthermore, the increasing emergence of high-computing-ability platforms oriented toward deep learning has made it possible to apply deep learning to interferometric signal processing in real time.

## Figures and Tables

**Table 1:** Comparison of signal processing methods for homodyne signals.

Method	Resolution	Bandwidth	Cost	Application
Error Calibration Methods	High	High	High	Academia
Ellipse Parameter Estimation Methods	High	High	Low	Industry/Academia

**Table 2:** Comparison of signal processing methods for heterodyne signals.

Method	Resolution	Bandwidth	Cost	Application
Pulse-Counting Methods	Medium	High	Low	Industry/Academia
Quadrature Phase-Locked Methods	High	High	Medium	Industry/Academia
Kalman Filtering Method	High	Medium	Medium	Academia

**Table 3:** Hardware platform comparison. Dev. stands for development and Dep. for deployment.

Platform	Power	Volume	Dev. Cost	Dev. Time	Dep. Cost
PCs	High	High	Low	Short	Medium
MCUs/DSPs	Low	Low	Low	Short	Low
FPGAs	Medium	Low	Low	Medium	Medium
ASICs	Low	Low	High	Long	Low

## Data Availability

The data presented in this study are available upon request from the corresponding author.
